# Abdominal Pain: an Uncommon Presentation of Myocardial Rupture

**DOI:** 10.36660/abc.20190495

**Published:** 2020-02

**Authors:** Daniel Seabra, Ana Neto, Inês Oliveira, Rui Pontes dos Santos, João Azevedo, Paula Pinto

**Affiliations:** 1 Centro Hospitalar do Tâmega e Sousa, Departamento de Cardiologia, Penafiel - Portugal

**Keywords:** Abdominal Pain, Myocardial Infarction/complications, Echocardiography, Doppler/methods, Thrombosis/surgery, Hypertension, Dyslipidemias, Tomography, X-Ray Computed/methods

**Figure 1:**
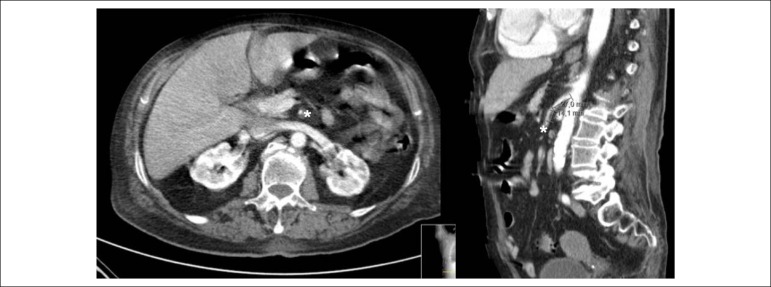
Abdominal computed tomography showing a thrombus in the superior mesenteric artery (white asterisk).

**Figure 2:**
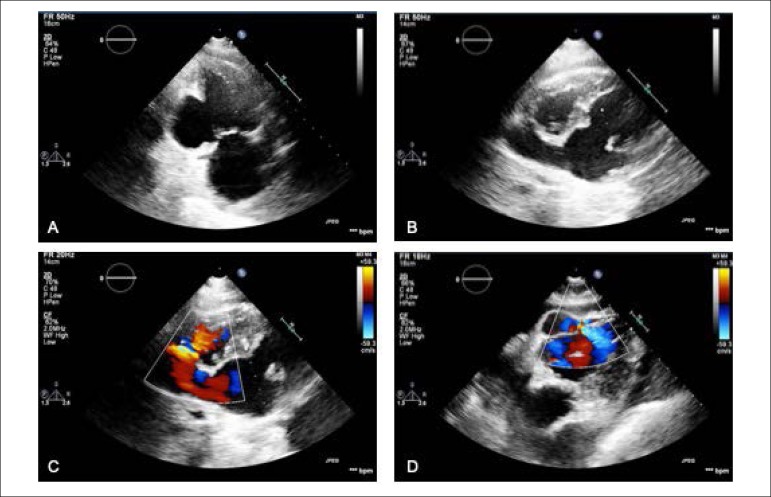
Pseudoaneurysm of left ventricular inferior wall on transthoracic echocardiography (TTE), apical two-chamber view (A). Left-right shunt in the basal segment of interventricular septum (B, C and D).

**Figure 3:**
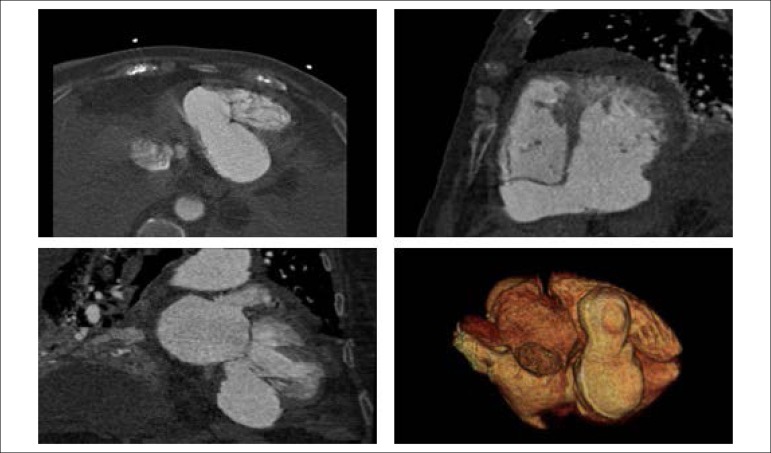
Cardiac computerized tomography showing contained myocardial rupture forming a pseudocavity that communicates with the true cavity of the right ventricle

An 84-years-old woman with hypertension and dyslipidemia was admitted in the emergency room with acute abdominal pain; patient complained of chest pain three weeks before hospital admission. On physical examination, she was hypotensive, tachycardic (with arrhythmic pulse), tachypneic and with diffuse abdominal pain. Electrocardiogram showed atrial fibrillation with rapid ventricular response, complete left bundle branch block and inferior Q waves. Abdominal computed tomography (CT) revealed a thrombus in the superior mesenteric artery (Figure 1, white asterisk). The patient showed a clinical course with congestive heart failure and low cardiac output. Transthoracic echocardiogram (Videos 1-2) showed mild left ventricular dilatation with a mild dysfunction and a pseudoaneurysm of the basal half of the posterior and inferior walls with left-to-right shunt, confirmed by color Doppler imaging (Figure 2 A-D). Cardiac CT (Video 3) revealed contained myocardial rupture, located at the basal segments of the inferior and posterior septal walls, extending to the free wall of the right ventricle, forming a pseudo-cavity, which communicates with the true cavity of the right ventricle (Figure 3). Despite vasopressor and inotropic support and proposal for cardiac surgery, the patient had an unfavorable course.

**Video 1:**
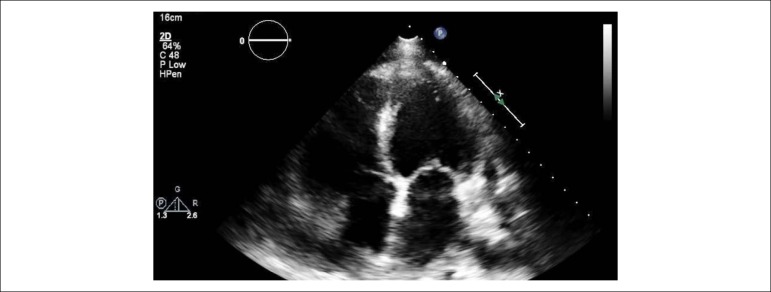
Transthoracic echocardiogram - apical windows. Link: http://publicacoes.cardiol.br/portal/abc/portugues/2020/v11402/dor-abdominal-uma-apresentacaoincomum-de-ruptura-miocardica.asp

**Video 2:**
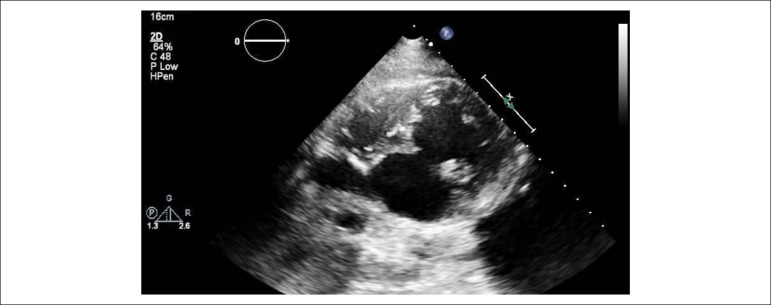
Transthoracic echocardiogram - modified subcostal window. Link: http://publicacoes.cardiol.br/portal/abc/portugues/2020/v11402/dor-abdominal-umaapresentacao-incomum-de-ruptura-miocardica.asp

**Video 3:**
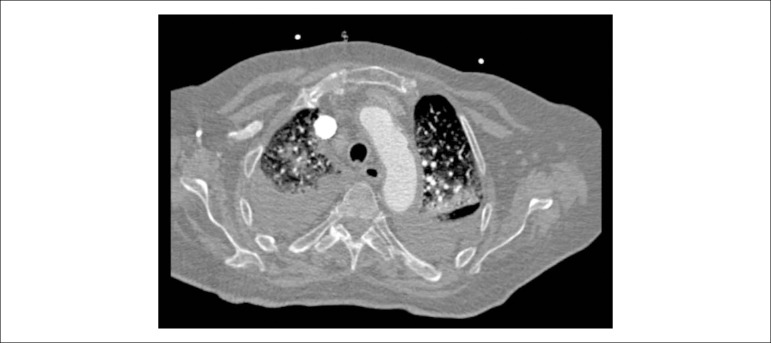
Thoracic CT scan .Link: http://publicacoes.cardiol.br/portal/abc/portugues/2020/v11402/dor-abdominal-uma-apresentacao-incomum-de-ruptura-miocardica.asp

Myocardial rupture demands a prompt diagnosis.^1^ Occurrence of late myocardial infarction should raise suspicion and clinical signs may be atypical.^2^

This case illustrates an interesting entity - pseudoaneurysm, with left-to-right shunt and contained myocardial rupture, which extended to the right ventricle, leading to a dismal prognosis.
